# The Influence of Storage Temperature on the Antibiotic Release of Vancomycin-Loaded Polymethylmethacrylate

**DOI:** 10.1155/2013/573526

**Published:** 2013-08-21

**Authors:** Dave W. Chen, Yuhan Chang, Pang-Hsin Hsieh, Steve W. N. Ueng, Mel S. Lee

**Affiliations:** ^1^Department of Orthopaedic Surgery, Linkou Chang Gung Memorial Hospital, Taiwan; ^2^Department of Orthopaedic Surgery, Chia Yi Chang Gung Memorial Hospital, Taiwan; ^3^Department of Medicine, College of Medicine, Chang Gung University, 6 West Sec. Chiapu Road, Putzu City, Chia Yi Hsien, Taiwan

## Abstract

Periprosthetic joint infection is devastating and increases medical expenditure and socioeconomic burden. Antibiotic-loaded cement spacer is useful in the interim period before the reimplantation surgery. Prefabricated antibiotic-loaded cement spacers can decrease operation time but have been limitedly used clinically. In the literature, there is no clear recommendation on the storage temperature for the prefabricated cement spacers. We used an in vitro model to analyze whether the storage temperature at 25°C, 4°C, or −20°C for 2 weeks or 3 months could affect the release of vancomycin from the cement. We found that the storage temperature and time had no significant effects on the pattern and amount of vancomycin release. The patterns of vancomycin release from the cement stored at different temperatures were similar with an abrupt release in the first 3 days and steadily declined in the following period. This study provides a preliminary result to justify the storage of fabricating antibiotic-loaded cement spacer sterilely packed at room temperature. Further studies to examine the effects of storage temperature on the mechanical strength and the release pattern of other antibiotics should be done to provide more evidence to support the clinical use of prefabricated ready-to-use antibiotic-loaded cement spacer.

## 1. Introduction

Periprosthetic joint infection (PJI) is a devastating condition that increases medical expenditure and patient's economic burden [1, 2]. For established PJI, the most accepted treatment modality is a two-stage reimplantation protocol [2]. During the interim period before reimplantation, antibiotic-loaded cement spacer has been widely adopted as an effective method to deliver high levels of local antibiotics for infection control and to maintain the soft tissue tension before the reimplantation surgery [3]. Antibiotic-loaded cement spacer is usually manufactured by surgeons during operation. This can be time consuming. If the causing bacteria are known preoperatively, the antibiotic-loaded cement spacer can be fabricated in advance. Prefabricated antibiotic-loaded cement spacer is appealing because it not only reduces operation time but also decreases blood loss [4–6]. Hailey et al. reported that the mechanical properties of bone cement stored at 37°C were more brittle than those stored at 21°C [7]. However, in the literature, there is no recommendation for the storage of antibiotic-loaded cement spacer. In our previous study, we found that the antibacterial activity of antibiotics in the bone cement could be maintained at −80°C [8]. But it is impractical to store and ship the cement spacer at −80°C in common clinical settings. We hypothesized that the storage temperatures of antibiotic-loaded cement had no significant influence on the antibiotic release. We tested our hypothesis by choosing room temperature (25°C), refrigerator (4°C), and freezer (−20°C) as the storage conditions by in vitro antibiotic release analysis.

## 2. Materials and Methods

Vancomycin 8 g (Gentle Pharmaceutical Co, Yunlin, Taiwan) was thoroughly mixed with 40 g of Surgical Simplex bone cement powder (Stryker Orthopaedics, Limerick, Ireland) in a stainless-steel container prior to the addition of the liquid monomer. After mixing with liquid monomer for 2 min with a doughy consistency, the cement mixture was pressed into plastic molds and cured at room temperature. The vancomycin-loaded cement discs were sterilely packed and divided into 3 groups with the storage temperature at 25°C, 4°C, and −20°C. The specimens were then divided into 2-week storage and 3-month storage.

After the completion of storage time, each cement disc (8 samples in each group) was immersed in polypropylene tube with 5 mL phosphate-buffered saline (PBS; pH 7.3) and agitated in an incubator at 37°C. Daily transfer of the cement disc into a new tube with PBS was continued for 28 days. The elution samples of 2 mL PBS at days 1, 3, 7, 14, and 28 were collected and stored at −80°C until analysis.

The concentration of vancomycin was determined using high-performance liquid chromatography (HPLC, model ALC 717, Waters Associates, Milford, MA, USA) with a stainless-steel column (RP18 column, 10 mm by 4.6 mm, 5 *μ*m particle size). The mobile phase consisted of water-acetonitrile 100 mM ammonium formate (composite ratio, 78/12/10). Accumulated amounts of vancomycin release from the cement discs were calculated.

Statistical analysis of repeated measure analysis of variance was used to determine differences in the vancomycin release between groups of different storage temperatures. A *P* value less than 0.05 was considered significant.

## 3. Results

**Figure 1:**
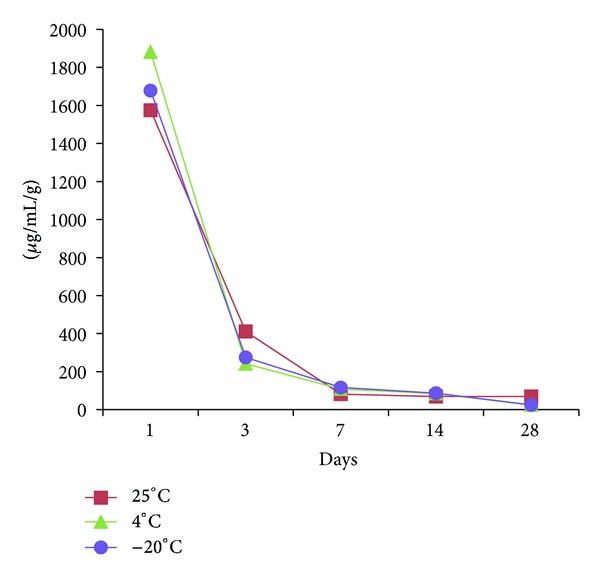
The release of vancomycin from samples stored at different temperatures for 2 weeks.

**Figure 2:**
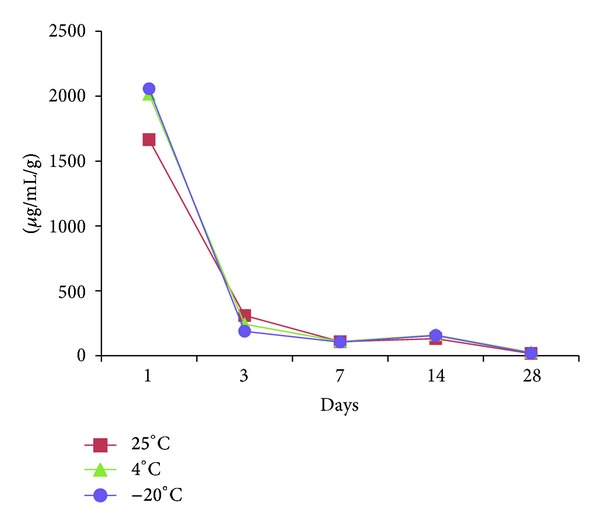
The release of vancomycin from samples stored at different temperatures for 3 months.

The patterns of vancomycin release from the cement discs stored at different temperatures were similar with an abrupt release in the first 3 days and steadily declined in the following period (Figures 1 and 2). The average weight of each cement disc was 3.94 g (range, 2.86 g to 4.75 g). The amount of vancomycin release from the cement discs was adjusted by their weight. The amount of vancomycin release on the first day was 1575 ± 96 *μ*g/mL/g, 1881 ± 116 *μ*g/mL/g, and 1678 ± 86 *μ*g/mL/g, respectively, with the storage temperatures at 25°C, 4°C, and −20°C for 2 weeks (mean ± standard deviation) (Figure 1). On the 14th day, it was 68 ± 10 *μ*g/mL/g, 85 ± 10 *μ*g/mL/g, and 86 ± 7 *μ*g/mL/g, respectively, at 25°C, 4°C, and −20°C. On the 28th day, it was 68 ± 2 *μ*g/mL/g, 24 ± 2 *μ*g/mL/g, and 24 ± 5 *μ*g/mL/g, respectively, at 25°C, 4°C, and −20°C. When the storage time was 3 months, the vancomycin release on the first day was 1665 ± 469 *μ*g/mL/g, 2014 ± 492 *μ*g/mL/g, and 2057 ± 598 *μ*g/mL/g, respectively, with the storage temperature at 25°C, 4°C, and −20°C (Figure 2). On the 14th day, it was 132 ± 6 *μ*g/mL/g, 160 ± 13 *μ*g/mL/g, and 156 ± 15 *μ*g/mL/g, respectively, at 25°C, 4°C, and −20°C. On the 28th day, it was 18 ± 2 *μ*g/mL/g, 26 ± 3 *μ*g/mL/g, and 20 ± 2 *μ*g/mL/g, respectively, at 25°C, 4°C, and −20°C. No difference could be found between the groups with different storage temperatures with 2 weeks or 3 months storage time.

The accumulated amount of vancomycin release from the each g of cement discs was, 29.17 mg, 28.23 mg, and 27.70 mg, respectively, when stored at 25°C, 4°C, and −20°C for 2 weeks. The antibiotic release ratios were 14.6%, 14.1%, and 13.8%, respectively. It was 30.45 mg (15.2%), 33.78 mg (16.9%), and 32.36 mg (16.2%), respectively, when stored at 25°C, 4°C, and −20°C for 3 months. There were no differences in terms of the accumulated vancomycin release from the cement discs stored at different temperature with 2 weeks or 3 months storage time.

## 4. Discussion

 Antibiotic-loaded cement spacer has been used for periprosthetic joint infection in the interim period to deliver local antibiotics while maintaining soft tissue tension and facilitating reimplantation surgery. The cement spacer can be articulating or nonarticulating depending on the surgeon's preference and the patient's condition. A PROSTALAC hip system is a commercial available spacer which consists of an all-polyethylene cemented acetabular component, a metal head, and a mold to construct antibiotic-loaded cement on a metal endoskeleton [3]. Reusable silicon, metal molds, or nonreusable plastic molds have also been fabricated with a metal endoskeleton for mechanical support. The PROSTALAC knee system has also been introduced with the femoral component incorporating metal runners and the tibial component incorporating inlay polyethylene plateaus [9]. The clinical success rates by using the antibiotic-loaded cement spacer are around 90% in the two-stage protocol [3, 9–11].

In clinical practice, a prefabricated antibiotic-loaded cement spacer is beneficial to patients with periprosthetic joint infection [4–6]. Severe PJI associated with sepsis can induce disseminated intravascular coagulopathy. Less severe PJI can also cause abnormal systemic coagulation problem [12]. In patients who have medical morbidities such as liver cirrhosis or coagulation abnormality, any measure to decrease the operation time and blood loss will be beneficial for the treatment of PJI [12–14]. Unfortunately, a pre-fabricated ready-to-use antibiotic-loaded cement spacer is not popular on the market because it is only available in some countries and the choice of antibiotics needs to be patient-specific according to the culture sensitivity results [4–6]. In chronic PJI, when the causative organisms are known, a prefabricated antibiotic-loaded cement spacer in sterile packing can facilitate surgery and save operation time. However, there is no recommendation or any guideline about the storage condition for antibiotic-loaded cement spacer. In our previous in vitro studies, we stored the antibiotic-cement specimens at −80°C and found that it would not affect the characteristics of antibiotics release from the cement as well as the bacterial killing abilities [8]. In this study, we examined the release of vancomycin from cement stored at 25°C (room temperature), 4°C (refrigerator), and −20°C (freezer). We found that the storage temperature did not affect the antibiotic release pattern and the daily or the accumulated amount of vancomycin released from the cement when the storage time was 2 weeks or 3 months. At 28 days, the concentration of vancomycin in the supernatant was still many folds higher than the minimal inhibitory concentration (MIC) for the bactericidal effects.

 This study provides a preliminary result to justify the practice of fabricating in-house antibiotic-loaded cement spacer sterilely packed and stored at room temperature before use. However, this result is limited and should not be translated to all clinical settings since only vancomycin was tested by using the in vitro model. In addition, the cement discs used in the study were not equal to the bulky cement spacer used clinically. Although the commercial available gentamicin-loaded cement beads (Septopal, Merck, Darmstadt, Germany) have been packed and shipped without temperature control since its introduction to the market, this study is the first report to examine the feasibility of storage condition of prefabricated antibiotic-loaded cement in terms of the antibiotic release.

 In summary, we found that the storage temperature at room temperature (25°C), refrigerator (4°C), or freezer (−20°C) of the antibiotic-loaded cement had no effect on the vancomycin release up to 3 months of the storage time. Further studies to examine the effects of storage temperature on the mechanical strength and the release pattern of other antibiotics should be done to provide more evidence to support the clinical use of prefabricated ready-to-use antibiotic-loaded cement spacer.
